# Atrial fibrillation as a contributing factor in the diagnostic algorithm for coronary subclavian steal syndrome and cardiac tamponade following coronary artery bypass graft surgery: a case study

**DOI:** 10.3325/cmj.2021.62.283

**Published:** 2021-06

**Authors:** Luka Perčin, Blanka Glavaš, Konja, Joško Bulum, Dražen Perkov, Majda Vrkić Kirhmajer

**Affiliations:** 1 Department of Cardiovascular Diseases, University Hospital Center Zagreb, Zagreb, Croatia; 2 Zagreb University School of Medicine, Zagreb, Croatia; 4 Department of Diagnostic and Interventional Radiology, University Hospital Center Zagreb, Zagreb, Croatia

## Abstract

Coronary subclavian steal syndrome (CSSS) is a complication of coronary artery bypass graft (CABG) surgery in patients with coexistent significant subclavian artery stenosis (SAS). It is characterized by a retrograde blood flow through the left internal mammary artery graft from the coronary to subclavian circulation, leading to myocardial ischemia. Current screening for CSSS includes bilateral blood pressure measurement for the detection of a significant inter-arm blood pressure difference. However, the commonly used automated sphygmomanometers have limited accuracy in patients with atrial fibrillation. Consequently, these patients are often underdiagnosed. We present a case of a 73-year-old man with a medical history of atrial fibrillation, peripheral artery disease, and CABG surgery four months before the current event, who came to the emergency department due to progressive dyspnea. The initial diagnostic management showed a large circulatory pericardial effusion, so the patient was admitted to the coronary care unit and underwent pericardial drainage. In the following days, due to a sudden high increase in cardiac troponin, the patient underwent an urgent coronary angiography, which revealed severe left SAS with functional CABG, indicating the occurrence of CSSS. Percutaneous transluminal angioplasty was then performed with an optimal angiographic result. The patient was discharged in good condition with adequate medicament therapy and instructions. This case report highlights atrial fibrillation as a contributing factor for the diagnosis of CSSS and pericardial tamponade after CABG surgery. Furthermore, we suggest a diagnostic approach that can reduce the incidence of both these severe complications.

Coronary subclavian steal syndrome (CSSS) occurs in the presence of subclavian artery stenosis (SAS) or occlusion and represents a reversal of blood flow in the left internal mammary artery (LIMA) bypass graft, which leads to coronary ischemia. It presents as a complication in 2.5–4.5% of patients undergoing coronary artery bypass graft (CABG) surgery. The prevalence is even higher in patients with peripheral artery disease (PAD), who have a 5-fold increased risk of SAS (1,2). It commonly presents as stable angina triggered by left upper extremity activity, but can also manifest as an acute coronary syndrome, acute heart failure, ventricular arrhythmia, or even sudden cardiac death (3). Digital subtraction angiography, the current gold standard in the imaging of CSSS, has lately been increasingly replaced by other diagnostic tools, such as duplex ultrasound (DUS), computed tomography angiography (CTA), and magnetic resonance angiography. The current guidelines recommend the endovascular approach as the first-line treatment of CSSS and vascular surgery as the second option (4).

## CASE REPORT

**Figure 1:**
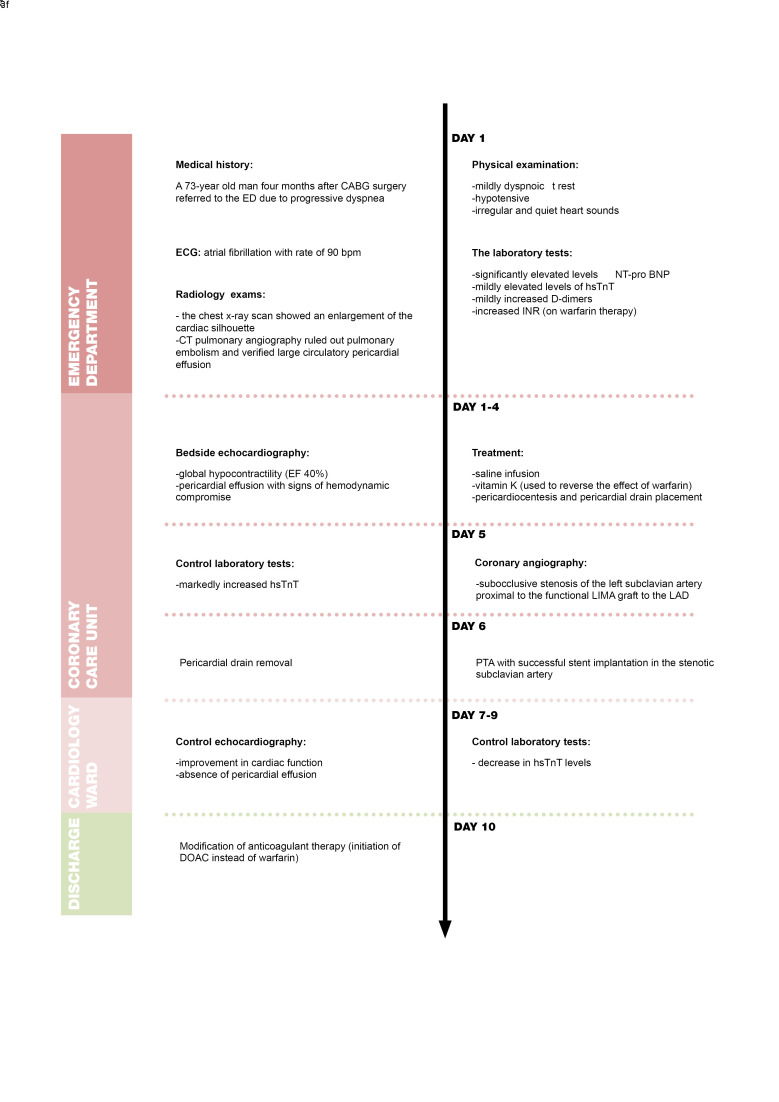
Timeline of the disease and treatment course. CABG – coronary artery bypass graft; ED – emergency department; EF – ejection fraction; ECG – electrocardiogram; CT – computed tomography; hsTnT – high-sensitive troponin; DOAC – direct oral anticoagulant; NT-pro BNP – N-terminal probrain natriuretic peptide; INR – international normalized ratio; LIMA – left internal mammary artery; LAD – left anterior descending artery; PTA – percutaneous transluminal angioplasty.

**Figure 2:**
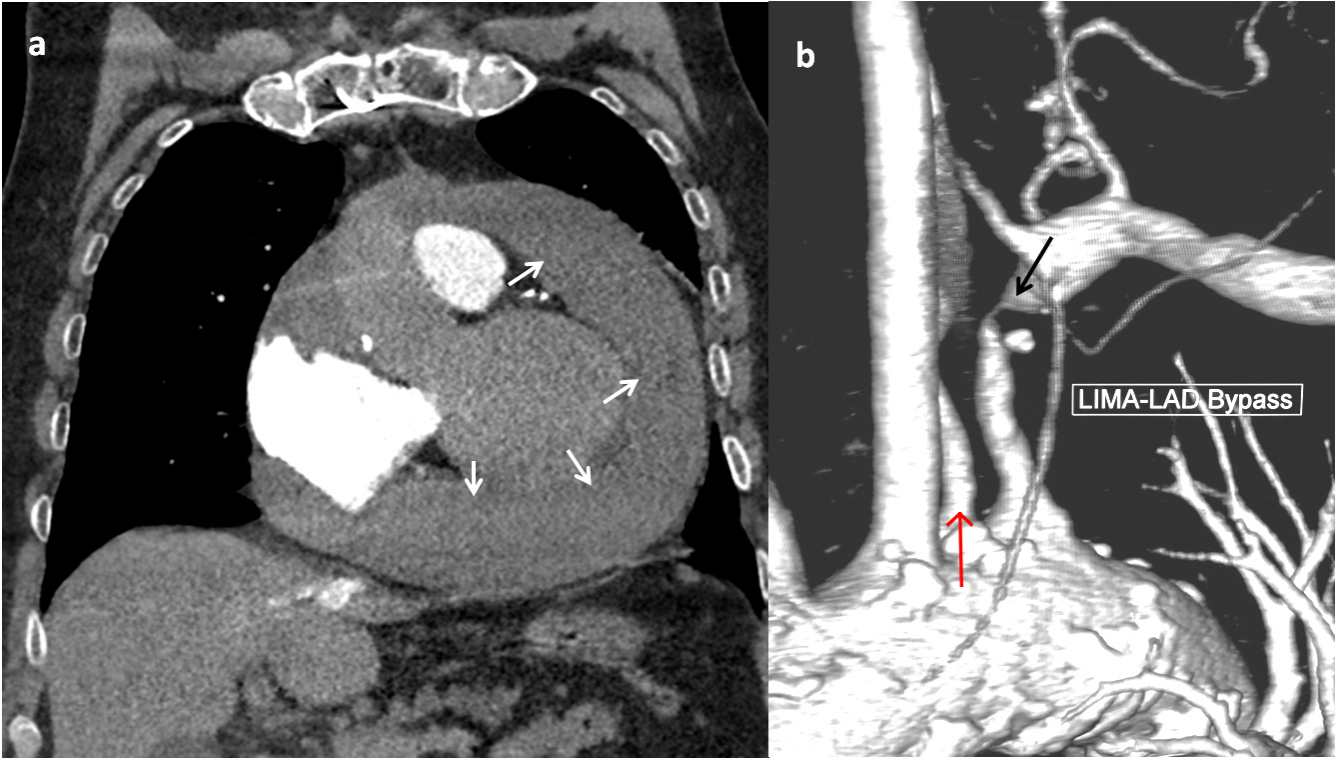
(**A**) Contrast-enhanced chest computed tomography (CT) image, multiplanar reconstruction, coronal plane, extensive pericardial effusion (white arrows). (**B**) CT angiography, 3D Volume Render image, high-grade stenosis of the left subclavian artery next to the left internal mammary artery (LIMA) bypass graft (black arrow), anomalous origin of the left vertebral artery from the aorta (red arrow).

**Figure 3:**
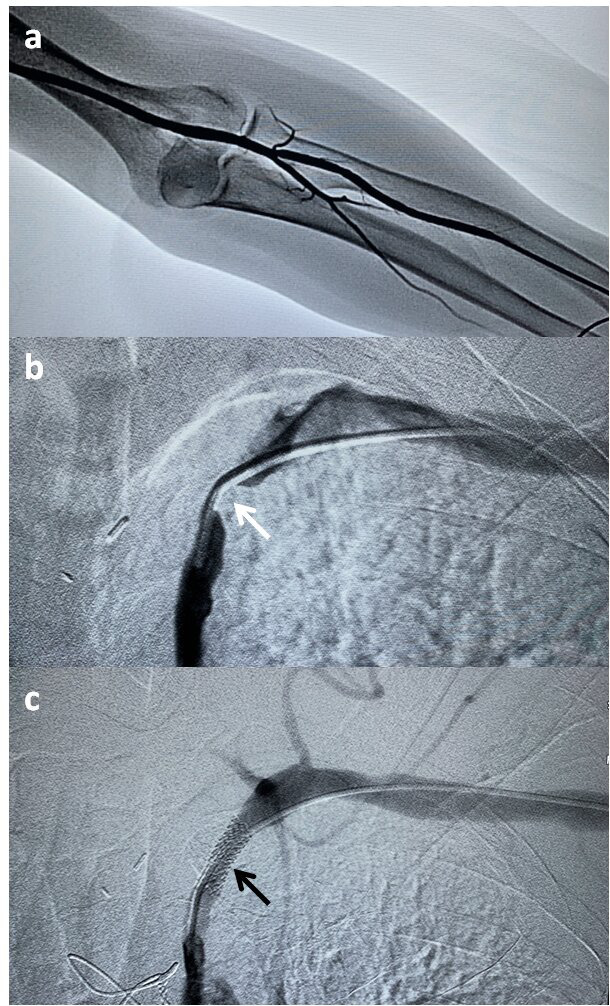
(**A**) Transradial digital subtraction angiography (DSA) access. (**B**) Selective DSA shows high-grade stenosis of the left subclavian artery next to the left internal mammary artery bypass graft (white arrow). (**C**) Selective DSA of the left subclavian artery shows successful dilation and stent implantation (black arrow).

We report on a case of a 73-year-old man who presented to our emergency department due to progressive shortness of breath (Figure 1). He was an ex-smoker (30 pack-years of cigarette smoking), with a medical history of dyslipidemia, arterial hypertension, atrial fibrillation, PAD, and left main coronary artery disease (CAD), which led to CABG surgery four months previously. His medical therapy included warfarin, antihypertensive drugs, statin, and a proton-pump inhibitor. Since surgery, he felt a gradual reduction in functional capacity with significant worsening of dyspnea in the last three days. On physical examination, the patient was mildly dyspnoic at rest and had low blood pressure (BP) readings of 105/74 mm Hg on the right arm and 97/69 mm Hg on the left arm. Heart sounds were quiet and irregular. Lung sound was clear bilaterally, with the exception of fine crackles in the right base. Jugular venous pressure was mildly elevated, and there was no evidence of peripheral edema. The rest of physical examination was unremarkable. Electrocardiogram (ECG) showed atrial fibrillation with a rate of 90 beats per minute (bpm), incomplete left bundle branch block, and diffuse T wave inversion in precordial leads. Laboratory tests demonstrated markedly elevated NT-proBNP levels of 3564 ng/L (reference levels <0.73 mg/L), mild increase in serial high sensitive troponin essays (hsTnT) of 31 ng/L and 51 ng/L (reference levels <14 ng/L), and normocytic anemia of 115 g/L (reference levels 138-175 g/L). The chest x-ray displayed an enlargement of the cardiac silhouette with mild pleural effusion and cranial redistribution of pulmonary vasculature, suggesting heart failure. Given the patient`s symptoms, CT pulmonary angiography was also requested to rule out pulmonary embolism. The test did not show signs of pulmonary embolus but showed a large circulatory pericardial effusion (Figure 2A). The patient was admitted to the coronary care unit. The bedside echo showed global hypocontractility of the left ventricle (ejection fraction of 40%) with a confirmed large circulatory pericardial effusion and increased respiratory variations in mitral and tricuspid inflow. Pericardiocentesis was performed with the drainage of 1500 mL of sanguineous effusion over a couple of days. The control laboratory tests showed an hsTnT increase (1865 ng/L) without the progression of clinical symptoms and without echographic or ECG changes. Coronary angiography showed significant stenosis of the left subclavian artery just proximal to the functional LIMA graft to the left anterior descending artery, which indicated the presence of CSSS. While the sudden hsTnT increase could have been explained by pericardiocentesis and consequential hemodynamic changes, myocardial strain, or mild myocardial trauma, it is likely that CSSS had significantly aggravated myocardial perfusion in specific hemodynamic circumstances following pericardiocentesis. Although subclavian bruit was absent, DUS of upper extremities showed reduced blood flow in the left arm, while CTA confirmed the previous angiographic finding and the anomalous origin of the left vertebral artery from the aorta (Figure 2B). As a result, percutaneous transluminal angioplasty was performed. A stent was implanted in the stenotic subclavian artery, followed by optimal blood flow across the treated vessel (Figure 3). The procedure's success was later also confirmed with control DUS. Control echocardiographic study revealed the absence of pericardial effusion and improvement in cardiac systolic function, which correlated with the patient` s clinical recovery. The patient was discharged from the hospital in a stable condition.

## DISCUSSION

In the modern era, improved life expectancy increased the prevalence of CABG surgeries, with LIMA as the most utilized conduit. This resulted in an overall higher number of postoperative adverse events. CSSS is a serious and potentially lethal complication with underestimated incidence, especially in some high-risk subgroups (2). Ever since it was first reported by Tyras and Barner (5), various clinical presentations of this syndrome resulting in severe outcomes have been described (1,3). For this reason, several author groups proposed diagnostic approaches to evaluate SAS and prevent this syndrome. For instance, the algorithm provided by Cua et al incorporated different diagnostic tests according to the patient`s risk factors such as PAD, arterial hypertension, dyslipidemia, and smoking (2). However, current SAS screening recommendations guidelines propose further investigation of SAS only after an inter-arm BP asymmetry of ≥15 mm Hg is found (4,6). Although bilateral blood pressure measurement is a valuable component of physical examination, it has a poor sensitivity in SAS detection. The absence of significant inter-arm BP variation could be explained by the presence of equal bilateral atherosclerosis or a development of extensive collaterals on the diseased side (2). Furthermore, automated sphygmomanometers have limited accuracy in patients with atrial fibrillation (7). The mean time between CABG surgery and the development of CSSS symptoms is 9.0 ± 8.4 years (8). In our patient, CSSS was diagnosed only four months after CABG, indicating that SAS might have been overlooked in the preoperative assessment. Since we documented equal preoperative bilateral BP, we assume that atrial fibrillation contributed to the diagnostic oversight of pre-existing unilateral SAS. Therefore, we suggest that patients with atrial fibrillation, especially those with concomitant risk factors, should undergo DUS of the upper extremities before CABG surgery, independently of their difference in bilateral brachial BP recordings.

In addition, postoperative pericardial effusion is a common complication of cardiac surgery, which can delay recovery. The effusion is often mild and clinically insignificant but can become large and life threatening, as was the case in our patient (9). Additionally, it has been reported that anticoagulants increase the risk of tamponade in patients who develop PE. Our case supports this observation as the patient was on warfarin therapy due to atrial fibrillation (10). Echocardiography is a well-utilized diagnostic tool in the assessment of pericardial effusion (9). Nevertheless, postoperative echocardiography is not always performed before hospital discharge after cardiac surgery. Consequently, pericardial effusion may often go unnoticed. In conclusion, we present this case to emphasize CSSS as a serious complication of CABG surgery and to highlight the need for SAS screening as a standard part of the preoperative evaluation in patients undergoing CABG surgery that includes LIMA, especially those with a medical history of both atrial fibrillation and PAD. Moreover, we also point out the importance of control echocardiographic study shortly after cardiac surgery, particularly in patients on anticoagulation therapy, in order to prevent potential complications such as cardiac tamponade.
